# Research Progress on Anti-Inflammatory and Antioxidant Mechanism of Artemether Based on MAPK/NF-κB Signaling Pathway

**DOI:** 10.3390/ijms27104607

**Published:** 2026-05-21

**Authors:** Mingxuan Yang, Kai Feng, Yanhong Li, Shuang Zeng, Hanwei Ma, Haijun Feng

**Affiliations:** 1 Clinical Medical School, Lanzhou University, Lanzhou 730000, China; dan0936@163.com (M.Y.); fengkai0828@163.com (K.F.); lyh702@126.com (Y.L.); 18394133532@163.com (S.Z.); mahanwei_xjtu@163.com (H.M.); 2 The College of Veterinary Medicine, Gansu Agricultural University, Lanzhou 730000, China

**Keywords:** artemether, MAPK/NF-κB pathway, inflammation-oxidative stress, Nrf2 signal, cross regulation

## Abstract

Artemether, a derivative of the natural compound artemisinin, is increasingly recognized for its multi-target anti-inflammatory and antioxidant properties. This review systematically elucidates the molecular mechanisms underlying these effects, focusing on artemether’s dual modulation of the MAPK/NF-κB and Nrf2 signaling pathways. We detail how artemether concurrently inhibits the MAPK/NF-κB axis—suppressing IKKβ phosphorylation and IκBα degradation to block NF-κB nuclear translocation—and downregulates p38/contextually modulates ERK phosphorylation. This leads to a significant reduction in key inflammatory mediators, including TNF-α, IL-6, and COX-2. Simultaneously, artemether activates the Nrf2 antioxidant pathway, upregulating HO-1 expression and enhancing the activity of SOD and GSH-Px, which effectively scavenges free radicals and reduces markers of oxidative damage such as MDA and 8-OHdG. The core therapeutic synergy arises from artemether’s disruption of the ROS-NF-κB positive feedback loop, which inhibits neutrophil infiltration and lipid peroxidation, thereby ameliorating tissue injury in experimental models of arthritis and neurodegenerative diseases. Compared to conventional NSAIDs and glucocorticoids, artemether exhibits a favorable safety profile, particularly regarding gastrointestinal effects, and demonstrates unique immunomodulatory potential. Future research directions should prioritize the development of nano-targeted delivery systems and the elucidation of pathway crosstalk at the single-cell level to advance the clinical translation of artemether for chronic inflammatory diseases.

## 1. Introduction

### 1.1. Chemical Properties and Natural Origin of Artemether

**Figure 1:**
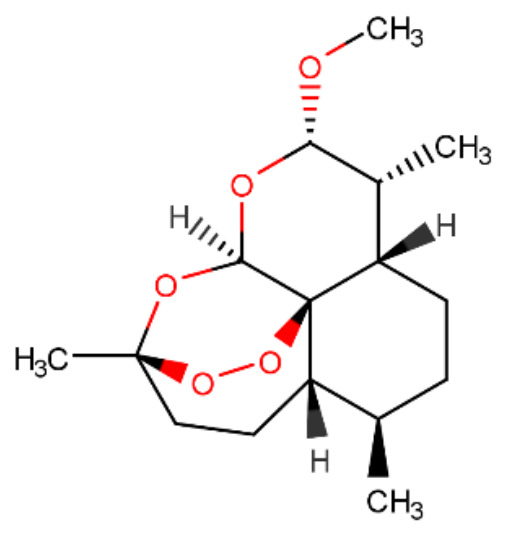
Structural formula of artemether.

Artemether (ARM) is a natural derivative with a sesquiterpenoid structure. Its chemical name is (3R,5aS,6R,8aS,9R,10S,12R,12aR)-decahydro-10-methoxy-3,6,9-trimethyl-3,12-epoxy-12H-pyrano [4,3-j]-1,2-benzodioxepin, with a molecular formula of C_16_H_26_O_5_ and a relative molecular mass of 298.37 [1]. Its core skeleton consists of an oxidatively rearranged 6/5/6/6 fused ring system containing a unique peroxide bridge (-O-O-) and an ether linkage. These structural features confer remarkable chemical stability [2,3], as illustrated in Figure 1. Under ambient conditions, artemether exists as white to pale-yellow crystals with a melting point range of 86 °C to 88 °C. It is readily soluble in organic solvents such as ethanol and chloroform but exhibits low water solubility [4]. Stability studies indicate that it can be stored for at least two years under light-protected and dry conditions; however, in strongly acidic or alkaline environments, hydrolysis may occur, resulting in cleavage of the peroxide bridge and the formation of secondary metabolites [2].

Regarding its natural origin, artemether is primarily extracted from the Asteraceae plant *Artemisia annua* L., which has a medicinal history spanning thousands of years in traditional medicine. Modern extraction techniques include solvent extraction, ultrasound-assisted extraction, and supercritical fluid extraction [5]. Artemether is synthesized through the chemical reduction and etherification of artemisinin (ART), with dihydroartemisinin (DHA) serving as an intermediate. Under catalysis by agents such as aluminum perchlorate, DHA is converted into artemether, and its β-isomer is the primary medicinal form due to its higher bioactivity [6]. In recent years, researchers have also isolated novel sesquiterpene dimers from related species such as Artemisia *ordosica*, further expanding the diversity of its natural sources [7].

In terms of traditional application, as a derivative of artemisinin, artemether has been a cornerstone of antimalarial therapy since the 1970s. Its mechanism of action relies on the reaction of the peroxide bridge with iron ions in the mitochondria of *Plasmodiumparasites*, generating reactive oxygen species (ROS) that damage the parasite’s membrane system and inhibit protein synthesis [8]. Clinical data indicate that its half-maximal inhibitory concentration (IC_50_) against chloroquine-resistant *Plasmodiumstrains* is six times lower than that of artemisinin, and it rapidly reaches peak plasma concentrations via oral or injectable administration [9]. According to World Health Organization (WHO) statistics, artemether-based combination therapies dominate the global antimalarial drug market, with an annual demand exceeding 1.5 billion US dollars [10].

Emerging research directions reveal that the sesquiterpene skeleton and functional groups of artemether confer multi-target pharmacological activities. For example, by inhibiting the NF-κB signaling pathway, it downregulates the expression of inflammatory mediators (e.g., COX-2, TNF-α), demonstrating significant anti-inflammatory effects in models of arthritis and enteritis. Its antioxidant properties stem from the electron delocalization capacity of the lactone ring, which enables the scavenging of free radicals and inhibition of lipid peroxidation, suggesting potential protective effects against neurodegenerative diseases [11]. In terms of antitumor activity, artemether induces apoptosis in cancer cells such as hepatocellular carcinoma (HepG2) and lung cancer (Huh7) by modulating the phosphoinositide 3-kinase (PI3K)/protein kinase B (AKT) pathway, with IC_50_ values of 54.8 μmol·L^−1^ and 31.4 μmol·L^−1^, while also suppressing tumor angiogenesis [12,13,14]. Furthermore, explorations of artemether in antiviral (e.g., HIV, influenza virus [15]) and antiparasitic (e.g., *Toxoplasma* [16]) fields have revealed its broad-spectrum therapeutic potential and novel mechanisms of action beyond malaria, thereby providing valuable insights for the development of multi-target drugs.

Research on the chemical properties and natural origins of artemether not only deepens the understanding of traditional medicines but also lays a molecular foundation for innovative cross-disciplinary applications. However, further elucidation is still required regarding the regulation of its metabolic pathways, the impact of environmental factors (e.g., temperature, humidity) on its stability, and the molecular network mechanisms underlying its pleiotropic effects.

### 1.2. Distinction Between Artemether and Other Artemisinin Derivatives: Scope of This Review

It is crucial to delineate the specific pharmacological context of artemether within the broader family of artemisinin derivatives, which includes the parent compound artemisinin, as well as dihydroartemisinin (DHA), artesunate, and arteether. While sharing the characteristic sesquiterpene lactone core with an endoperoxide bridge, these derivatives possess distinct physicochemical properties due to modifications at the C-10 or C-16 positions. These differences confer unique pharmacokinetic profiles, including variations in solubility, metabolic stability, and the rate of conversion to the active metabolite DHA. Consequently, their biological activities, including anti-inflammatory and antioxidant potencies, may not be directly equivalent. A recognized limitation in the existing literature is the occasional extrapolation of mechanistic findings from one derivative to another without direct experimental verification. To ensure precision, this review intentionally focuses on and cites evidence specifically derived from studies where artemether was the direct experimental intervention. When discussing broader mechanisms or for comparative context, findings from studies utilizing other derivatives (e.g., artemisinin, artesunate) are explicitly acknowledged as such. This approach aims to provide a clear and accurate synthesis of the evidence base specifically pertaining to artemether, while acknowledging the shared pharmacological framework of its analogues. Future research directly comparing the efficacy and detailed mechanisms of different artemisinin derivatives in models of inflammation and oxidative stress is warranted to clarify their relative therapeutic potentials.

### 1.3. Pathological Link Between Inflammation and Oxidative Stress

The therapeutic potential of artemether in chronic diseases is closely linked to its ability to intervene in the pathological interplay between inflammation and oxidative stress, which is driven by interconnected molecular pathways. Inflammation and oxidative stress are core drivers in multiple pathological processes. They form a vicious cycle through complex molecular networks, accelerating tissue damage and disease progression. Studies have shown that the excessive production of reactive oxygen species (ROS) is not only a direct marker of oxidative stress but can also trigger a cascade release of pro-inflammatory cytokines (e.g., TNF-α, IL-6, IL-1β) by activating signaling pathways such as NF-κB and MAPK, thereby amplifying the inflammatory response. For instance, ROS generation induced by PM2.5 exposure can significantly upregulate the expression of inflammatory factors by activating the NF-κB and MAPK pathways, leading to pulmonary and cardiovascular system damage [17]. In zinc deficiency models, oxidative stress, through the phosphorylation of IκBα and NF-κB p65, activates MAPK branches (ERK/c-Jun N-terminal kinase (JNK)/p38), ultimately triggering renal inflammation and apoptosis [18]. This bidirectional regulatory mechanism reveals the close molecular-level connection between oxidative stress and inflammation.

**Figure 2:**
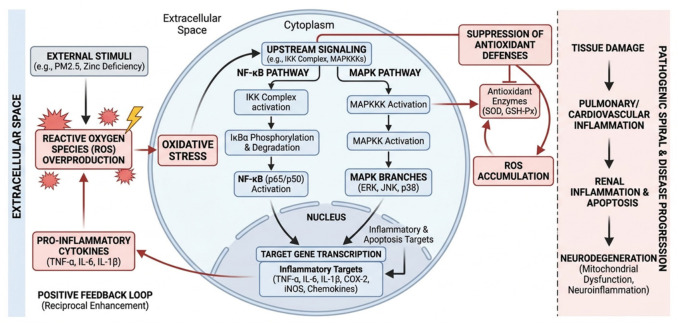
Molecular mechanism and positive feedback loop of ROS-induced NF-κB/MAPK signaling pathway mediating the interplay between inflammation and oxidative stress. Created in BioRender. Yang, M. (2026) https://BioRender.com/2nsn9cx (accessed on 18 May 2026).

The MAPK/NF-κB signaling pathways play a dual role in this process (Figure 2): on the one hand, the MAPK family (ERK, JNK, p38) regulates the expression of inflammatory genes by phosphorylating transcription factors and mediates the balance between apoptosis and proliferation [19,20]; on the other hand, NF-κB acts as a “molecular hub” for the inflammatory response by regulating hundreds of target genes, including COX-2, inducible nitric oxide synthase (iNOS), and chemokines [21]. It is noteworthy that oxidative stress not only directly activates these pathways but can also form a positive feedback loop by inhibiting the activity of antioxidant enzymes (e.g., SOD, GSH-Px), thereby weakening the cell’s ability to clear ROS [22]. For example, in neurodegenerative diseases, ROS-induced NF-κB activation can promote neuroinflammation, and the sustained inflammatory microenvironment further exacerbates mitochondrial dysfunction and oxidative damage [23,24].

**Figure 3:**
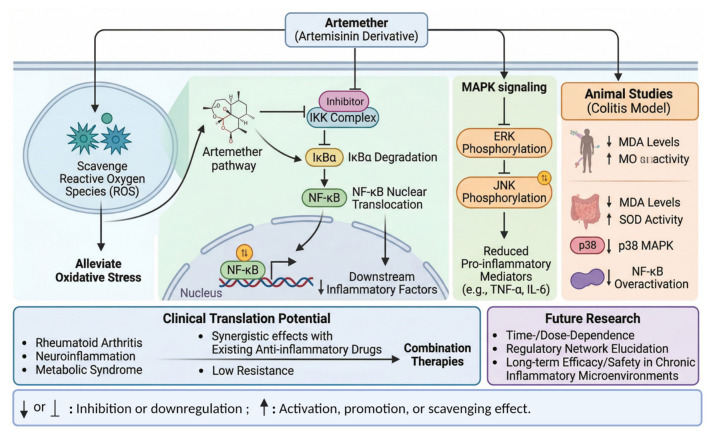
Mechanism of artemether’s multi-target intervention on the NF-κB/MAPK pathway to block the vicious cycle between inflammation and oxidative stress. Created in BioRender. Yang, M. (2026) https://BioRender.com/gvgqie2 (accessed on 18 May 2026). Note: The schematic representation primarily summarizes artemether’s inhibitory effects on the MAPK/NF-κB pathway observed in classic inflammatory models. It is important to note that the regulatory effect of artemether on MAPK signaling, particularly ERK, is highly integrated with the pathological context. As detailed in Section 4.2.2, artemether can activate the ERK pathway to confer neuroprotection in cerebral ischemia models [25]. This apparent duality underscores its role as a network modulator rather than a unidirectional inhibitor, a concept mechanistically elucidated later in this review.

In light of this pathological link, the regulatory role of artemether demonstrates unique scientific value, as shown in Figure 3. As a derivative of artemisinin, artemether not only alleviates oxidative stress by scavenging ROS but also specifically inhibits the activity of the inhibitor kappa B kinase (IKK) complex, thereby blocking IκBα degradation, which subsequently suppresses NF-κB nuclear translocation and the release of downstream inflammatory factors [25,26]. Its modulation of the MAPK pathway appears to be context-dependent [27]. While a substantial body of evidence indicates that artemether can inhibit the phosphorylation of p38 MAPK and ERK1/2 in classic inflammatory disease models (e.g., arthritis, colitis, and osteoclastogenesis), thereby reducing the expression of pro-inflammatory mediators (e.g., TNF-α, IL-6) [25,28,29,30], contrasting findings in neuroprotection models demonstrate that artemether stimulates the phosphorylation of ERK1/2 to confer cytoprotective effects [25]. This highlights the compound’s pleiotropic and potentially cell type- or disease state-specific regulatory effects on signaling networks. Animal studies have shown that artemether can significantly decrease malondialdehyde (MDA) levels and restore superoxide dismutase (SOD) activity in colitis models, while also inhibiting the overactivation of the p38 MAPK and NF-κB pathways [31]. This multi-target regulatory characteristic endows it with broad potential for clinical translation in treating inflammation-related diseases, such as rheumatoid arthritis, neuroinflammation, and metabolic syndrome. Furthermore, the synergistic effects and low resistance profile of artemether in combination with existing anti-inflammatory drugs provide a theoretical basis for developing novel combination therapies. Future research should further elucidate the time- and dose-dependence of its regulatory network and explore its long-term efficacy and safety within chronic inflammatory microenvironments.

### 1.4. Literature Search Strategy

A systematic literature search was conducted to identify all relevant preclinical studies on the anti-inflammatory and antioxidant mechanisms of artemether related to the MAPK/NF-κB pathways. Electronic databases, including PubMed, Web of Science, and Google Scholar, were searched from their inception until December 2025. The search strategy employed a combination of keywords and Medical Subject Headings (MeSH) terms: “artemether,” “anti-inflammatory,” “antioxidant,” “oxidative stress,” “MAPK,” “NF-kappa B,” “Nrf2,” and “signaling pathway,” connected with Boolean operators (AND, OR). The inclusion criteria were: (1) original research articles in English; (2) in vitro or in vivo models; (3) artemether as the direct experimental intervention; and (4) investigation of inflammatory/antioxidant outcomes or the specified pathways. Exclusion criteria encompassed studies using only other artemisinin derivatives (e.g., artemisinin, artesunate) without a direct artemether control group, and research focused solely on antimalarial activity. The screening process involved an initial title/abstract review, followed by a full-text assessment of potentially eligible articles. Data on the model, artemether dosage, interventions, key findings, and references were extracted from the included studies to construct this narrative review.

## 2. Biological Functions of the MAPK/NF-κB Signaling Pathway

### 2.1. Basic Structure and Function of the Pathway

#### 2.1.1. Structure and Function of the MAPK Pathway

**Figure 4:**
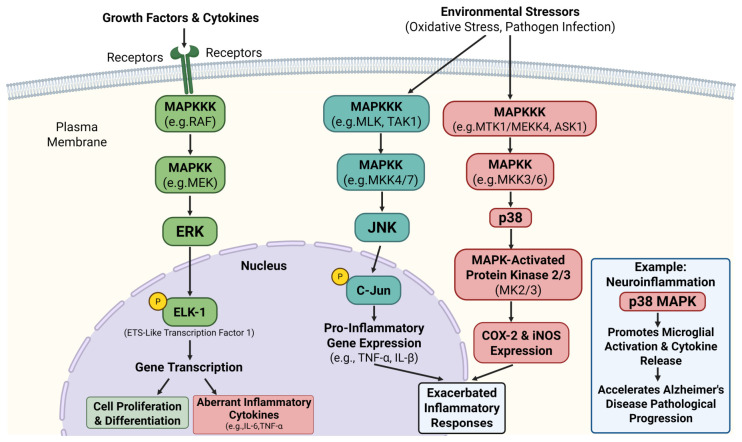
The three-tiered kinase cascade structure of the MAPK signaling pathway and the differential functions of its three core branches (ERK/JNK/p38) in inflammatory responses. Created in BioRender. Yang, M. (2026) https://BioRender.com/607ksl9 (accessed on 18 May 2026).

The MAPK signaling pathway consists of a three-tiered kinase cascade (mitogen-activated protein kinase kinase kinase, MAPKKK → mitogen-activated protein kinase kinase, MAPKK → MAPK) and encompasses three core branches (as shown in Figure 4): ERK, JNK, and p38 [19]. The ERK pathway primarily responds to growth factor and cytokine stimulation. It promotes cell proliferation and differentiation by phosphorylating transcription factors (e.g., Ets-like transcription factor 1, Elk-1). However, its overactivation can lead to aberrant release of inflammatory cytokines (e.g., IL-6, TNF-α) [20,32]. The JNK and p38 pathways, in contrast, are preferentially activated by environmental stressors such as oxidative stress and pathogen infection. JNK regulates the expression of pro-inflammatory genes (e.g., TNF-α, IL-1β) by phosphorylating c-Jun [33], while p38 enhances the expression of COX-2 and iNOS by activating MAP kinase-activated protein kinase 2/3 (MK2/3) kinases, thereby exacerbating inflammatory responses. For instance, in neuroinflammation, p38 MAPK accelerates the pathological progression of Alzheimer’s disease by promoting microglial activation and cytokine release [34,35].

#### 2.1.2. Fundamental Regulatory Mechanisms of the NF-κB Pathway

**Figure 5:**
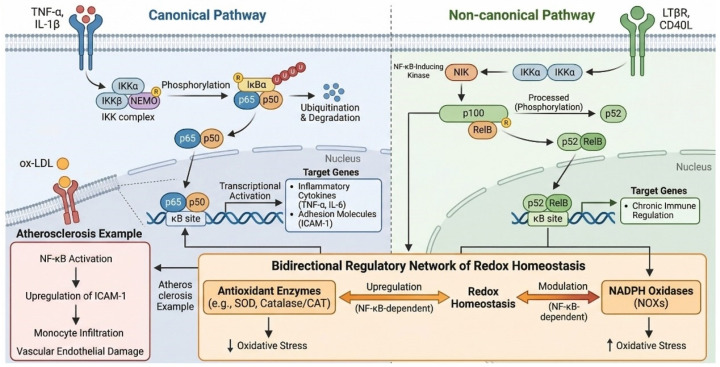
Dual activation mechanisms (canonical/non-canonical) and functions of the NF-κB signaling pathway. Created in BioRender. Yang, M. (2026) https://BioRender.com/0nm8mmv (accessed on 18 May 2026).

As shown in Figure 5, the NF-κB pathway regulates genes associated with inflammation and oxidative stress through canonical and non-canonical pathways. The canonical pathway relies on the phosphorylation and degradation of IκBα by the IKK complex, which releases NF-κB dimers (p50/p65). These dimers translocate to the nucleus and directly bind to κB sites on genes such as TNF-α and IL-6 [36]. The non-canonical pathway is mediated by NF-κB-inducing kinase (NIK), which processes p100 into p52. The p52 subunit then forms dimers with RelB to participate in the immune regulation of chronic inflammation. Furthermore, NF-κB contributes to a bidirectional regulatory network of redox homeostasis by modulating the expression of antioxidant enzymes (e.g., SOD, catalase, CAT) and nicotinamide adenine dinucleotide phosphate (reduced form, NADPH) oxidases. For example, in atherosclerosis, oxidized low-density lipoprotein (ox-LDL) upregulates intercellular adhesion molecule-1 (ICAM-1) by activating NF-κB, thereby promoting monocyte infiltration and vascular endothelial damage [37].

#### 2.1.3. Experimental Modulation of NF-κB by Artemether

The schematic in Figure 5 illustrates the canonical and non-canonical NF-κB pathways. It is crucial to interpret this diagram in the context of experimental findings on artemether. Current evidence indicates that artemether exerts its anti-inflammatory effects primarily through targeted intervention in the canonical pathway. Specifically, as denoted conceptually in Figure 5, artemether has been shown to inhibit the phosphorylation and degradation of IκBα, thereby sequestering the NF-κB p65/p50 dimer in the cytoplasm and blocking its nuclear translocation [38]. This molecular action effectively suppresses pro-inflammatory gene transcription. Supporting evidence from disease models further confirms the inhibition of the NF-κB pathway by artemether [31]. In contrast, direct experimental evidence for artemether modulating key components of the non-canonical pathway (e.g., NIK-mediated processing of p100) remains to be established. Therefore, the absence of modulation symbols in the non-canonical section of Figure 5 accurately reflects the current state of evidence, distinguishing established actions from potential but unverified targets.

### 2.2. Interaction Between Inflammation and Oxidative Stress

#### 2.2.1. Interaction Mechanisms of Inflammatory Mediators and Oxidative Stress

**Figure 6:**
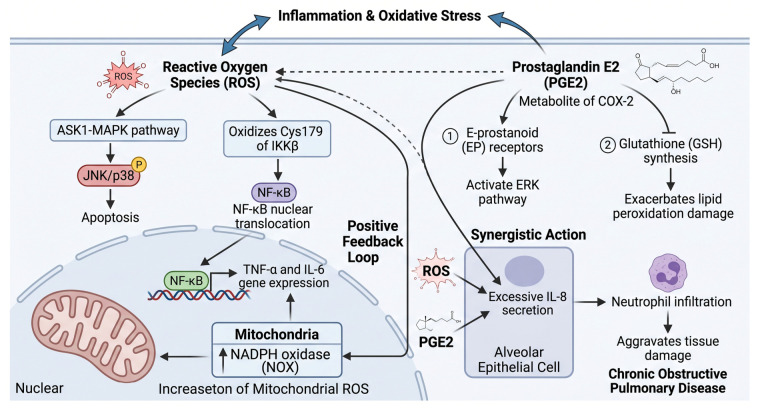
ROS and PGE2 as key mediators driving the cross-talk and positive feedback loop between inflammation and oxidative stress. Created in BioRender. Yang, M. (2026) https://BioRender.com/x24d58t (accessed on 18 May 2026).

Reactive oxygen species (ROS) and prostaglandin E2 (PGE2) are key mediators in the interplay between inflammation and oxidative stress. As shown in Figure 6, ROS enhance the phosphorylation of JNK/p38 by activating the apoptosis signal-regulating kinase 1 (ASK1)-MAPK pathway [39], while simultaneously oxidizing the cysteine 179 (Cys179) residue of IKKβ to promote NF-κB nuclear translocation. Conversely, NF-κB-induced TNF-α and IL-6 increase mitochondrial ROS production by upregulating the activity of NADPH oxidase (NOX), establishing a positive feedback loop [40]. PGE2, a metabolite of COX-2, not only activates the ERK pathway via E-prostanoid (EP) receptors but also exacerbates lipid peroxidation damage by inhibiting glutathione (GSH) synthesis [41,42]. It is noteworthy that Figure 6 illustrates this fundamental pathological interplay between inflammation and oxidative stress. The specific multi-target intervention of artemether in disrupting this vicious cycle is detailed in Figure 3. For example, in chronic obstructive pulmonary disease, the synergistic action of ROS and PGE2 leads to excessive secretion of IL-8 by alveolar epithelial cells, which attracts neutrophil infiltration and aggravates tissue damage [43].

#### 2.2.2. Pathological Significance of Pathway Dysregulation in Chronic Inflammatory Diseases

Aberrant activation of the MAPK/NF-κB signaling pathways is a common pathological feature in various chronic inflammatory diseases. In rheumatoid arthritis, persistently activated p38 MAPK promotes joint destruction by enhancing matrix metalloproteinase-9 (MMP-9) expression, while NF-κB-driven excessive secretion of IL-6 leads to an imbalance between T helper 17 (Th17) and regulatory T (Treg) cells [44]. During the progression of atherosclerosis, oxidized low-density lipoprotein (ox-LDL) activates the ERK/NF-κB axis via the lectin-like oxidized LDL receptor-1 (LOX-1) receptor, promoting vascular endothelial ICAM-1 expression and monocyte infiltration. Concurrently, ROS-induced mitochondrial DNA damage further exacerbates foam cell formation. Furthermore, in inflammatory bowel disease, overactivation of the JNK signaling pathway disrupts intestinal barrier integrity by promoting apoptosis and fibrosis of intestinal epithelial cells [45,46].

#### 2.2.3. Targeted Regulation Strategies

Therapeutic strategies targeting the interactive nodes of the MAPK/NF-κB pathways have become a research focus. For instance, IKKβ inhibitors can block the nuclear translocation of NF-κB, while JNK-specific small interfering RNA (siRNA) reduces the release of pro-inflammatory factors by inhibiting c-Jun phosphorylation [47]. Natural compounds, such as myricetin, significantly lower IL-6 and TNF-α levels in hepatic ischemia-reperfusion injury by inhibiting the MAPK/NF-κB pathways [48]. Furthermore, the combined application of targeted ROS scavengers (e.g., N-acetylcysteine) and anti-inflammatory drugs can synergistically alleviate the vicious cycle of oxidative stress and inflammation [49].

The MAPK/NF-κB pathways occupy a central position in the interactive regulation of inflammation and oxidative stress, and their dysregulation is a key driving factor in the progression of chronic inflammatory diseases. Future research should focus on further elucidating the spatiotemporal dynamic interactions between these pathways and developing multi-target agents to overcome the limitations of single-pathway therapy. For example, employing single-cell sequencing technology to reveal the heterogeneous activation patterns of these pathways in different cell subsets may provide novel insights for precision medicine.

### 2.3. Activation of MAPK/NF-κB by Canonical Inflammatory Stimuli

Research has demonstrated that stimulation of macrophages with lipopolysaccharide (LPS) via the Toll-like receptor 4 (TLR4) activates key downstream signaling pathways. The LPS signal is transduced primarily through the adapter protein MyD88. This leads to the activation of the kinase Transforming Growth Factor-β-activated kinase 1 (TAK1). Activated TAK1 subsequently phosphorylates and activates Mitogen-Activated Protein Kinase Kinases (MKKs), which in turn induce the phosphorylation and activation of Mitogen-Activated Protein Kinases (MAPKs) such as p38 MAPK, JNK, and ERK1/2. In parallel, the same signaling axis activates the IκB kinase (IKK) complex via TAK1. The activated IKK complex then phosphorylates the NF-κB inhibitor IκBα, targeting it for ubiquitination and rapid proteasomal degradation. The degradation of IκBα releases its cytoplasmic sequestration of the NF-κB transcription factor complex, predominantly the p50/RelA dimer, allowing its nuclear translocation. These coordinated events—MAPK activation and the triggering of the NF-κB signaling cascade—collectively drive the transcriptional initiation of numerous pro-inflammatory cytokines, chemokines, and immunomodulatory molecules [21].

## 3. Anti Inflammatory Mechanism of Artemether and Its Regulation of the MAPK/NF-κB Pathway

**Figure 7:**
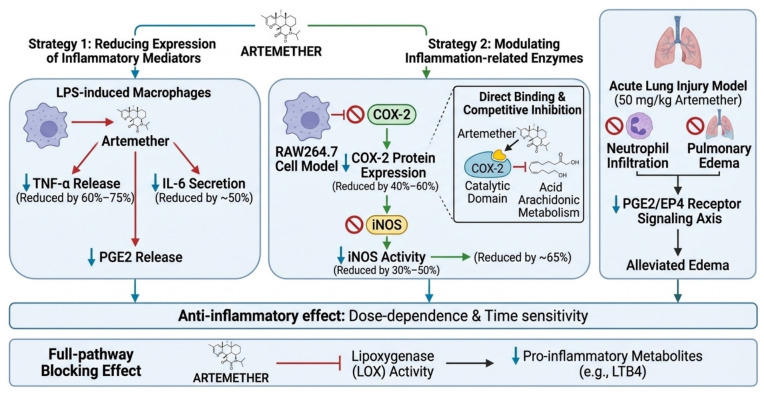
Anti-inflammatory and antioxidant mechanisms of artemether via the MAPK/NF-κB axis. Created in BioRender. Yang, M. (2026) https://BioRender.com/i5h2tvl (accessed on 18 May 2026).

As an important derivative of artemisinin, the anti-inflammatory effects of artemether have been validated through multidimensional experimental studies. Its core mechanism involves the suppression of inflammatory mediator release and targeted regulation of the MAPK/NF-κB signaling pathway. This section, based on advances in molecular biology and pharmacological research, systematically elaborates the multi-level molecular basis of its anti-inflammatory action. The underlying mechanisms are illustrated in Figure 7.

### 3.1. Molecular Basis of Anti-Inflammatory Effects

Artemether inhibits inflammatory responses through a dual strategy: on the one hand, it reduces the expression levels of key inflammatory mediators, and on the other hand, it modulates the activity of inflammation-related enzymes. Studies have shown that artemether can significantly decrease the release of TNF-α, IL-6, and prostaglandin E2 (PGE2) in lipopolysaccharide (LPS)-induced inflammatory cells. In LPS-activated BV2 microglial cells, artemether (5–40 μM) treatment dose-dependently reduced the production of TNF-α, IL-6, and PGE2, while also suppressing the protein expression of inducible nitric oxide synthase (iNOS) and cyclooxygenase-2 (COX-2) [38]. This inhibitory effect on inflammatory mediators is closely associated with artemether’s interference with the arachidonic acid metabolic pathway and downstream signaling.

### 3.2. Direct Regulation of the MAPK/NF-κB Pathway

The anti-inflammatory action of artemether is rooted in its direct interference with key nodal points of the MAPK/NF-κB signaling cascades. Contrary to a generalized pathway description, the following synthesis is strictly based on experimental evidence where artemether was the direct intervention and its effect on specific phospho-proteins, nuclear translocation, or DNA-binding activity was explicitly measured. This evidence-based delineation aims to establish a clear mechanistic link between artemether and the inflammatory signaling network.

#### 3.2.1. Regulatory Mechanism of the NF-κB Pathway

Artemether exerts its anti-inflammatory effects through a multi-target mechanism, with a critical component being the interference with the canonical NF-κB signaling cascade. This action is mediated, importantly, via the activation of the Nrf2 antioxidant pathway [31,38]. Specifically, artemether inhibits the phosphorylation and subsequent degradation of IκBα, thereby sequestering the NF-κB p65 subunit in the cytoplasm and blocking its nuclear translocation, as demonstrated by immunofluorescence and cellular fractionation assays. Electrophoretic mobility shift assays (EMSA) provide direct functional corroboration, showing that artemether treatment significantly reduces the DNA-binding capacity of NF-κB in activated microglia. Crucially, mechanistic studies using siRNA knockdown have established that this suppression of NF-κB activity and the consequent downregulation of pro-inflammatory genes (e.g., TNF-α, IL-6, COX-2) are dependent on Nrf2 activation [38]. It is noteworthy that the regulatory effect of artemether on inflammatory signaling exhibits context-dependence; for instance, in RANKL-induced osteoclastogenesis, it specifically inhibits the MAPK pathway without significantly affecting NF-κB activation [50].

#### 3.2.2. Inhibitory Effects on the MAPK Signaling Cascade

In terms of MAPK pathway regulation, artemether demonstrates broad inhibitory effects on key MAPK family members. In RANKL-induced osteoclastogenesis, artemether (10 μM) significantly suppressed the phosphorylation of ERK, JNK, and p38 MAPK in RAW264.7 cells, without affecting the NF-κB and PI3K/Akt pathways under the same conditions [50]. This inhibition occurred at the level of upstream kinases MEK1/2, MKK3/6, and MKK7 [50]. Furthermore, in LPS-activated BV2 microglia, artemether was shown to inhibit p38 MAPK signaling, contributing to its anti-inflammatory effects [38]. This broad suppression of MAPK activation leads to the downregulation of downstream transcription factors (such as c-Fos and NFATc1 in osteoclasts) and inflammatory mediators, thereby alleviating inflammatory tissue damage [50].

The collective direct molecular evidence presented above, which delineates artemether’s inhibition of IKK/IκBα phosphorylation, NF-κB nuclear translocation, and MAPK (p38, JNK, and contextually, ERK) activation, is integrated and visually summarized in Figure 3 and Figure 5. These schematics highlight the multi-target intervention of artemether within the inflammatory signaling network, based on the experimental findings cited herein. It is critical to reconcile this with the established activating effect of artemether on the ERK pathway in models of neuroprotection, as discussed in Section 4.2.2, which highlights the compound’s context-defined signaling outcomes.

#### 3.2.3. Pathway Interactions and Synergistic Effects

The regulation of the MAPK/NF-κB pathways by artemether does not occur in isolation; it is intricately linked to the activation of endogenous antioxidant pathways, particularly the nuclear factor erythroid 2-related factor 2 (Nrf2)/antioxidant response element (ARE) axis. A key mechanism of artemether’s action is the activation of Nrf2, leading to the upregulation of cytoprotective enzymes such as heme oxygenase-1 (HO-1) [38]. Notably, the anti-inflammatory effects of artemether, including the suppression of TNF-α, IL-6, PGE2, iNOS, and COX-2 in LPS-activated microglia, are mediated through Nrf2-dependent mechanisms, as Nrf2 knockdown by siRNA reverses these inhibitory effects [38]. This crosstalk between Nrf2 activation and NF-κB/MAPK inhibition represents a crucial multi-target synergistic action, granting artemether unique advantages in combating chronic inflammatory diseases. Future research should integrate single-cell sequencing and proteomic technologies to systematically elucidate the multidimensional regulatory network of artemether within the inflammatory microenvironment.

The collective evidence delineates a compelling picture of artemether as an inhibitor of the MAPK/NF-κB axis. However, a critical appraisal necessitates acknowledging the context upon which this conclusion rests. First, the robustness of this inhibitory claim varies; while the suppression of p38/JNK and NF-κB appears consistent across multiple inflammatory models, the effect on ERK1/2 is paradigmatic of the compound’s context-dependent actions. As highlighted in Section 1.3, artemether can activate ERK1/2 to confer neuroprotection, revealing that its designation as a simple ‘MAPK inhibitor’ is an oversimplification. Second, many mechanistic studies rely on pharmacologic inhibitors or knockdown approaches to establish pathway necessity. Future research employing cell-type-specific genetic models would strengthen the causal linkage between artemether’s binding targets and its observed anti-inflammatory phenotypes.

## 4. Antioxidant Mechanisms of Artemether and Cross-Talk with Signaling Pathways

The antioxidant effects of artemether are not only reflected in its ability to directly scavenge free radicals but also in its multi-dimensional synergistic effects achieved by modulating redox homeostasis-related signaling pathways and intertwining with anti-inflammatory mechanisms. This section systematically elaborates the molecular basis of its antioxidant activity and its cross-regulatory mechanisms with inflammatory pathways, based on in vitro experiments and animal model data.

### 4.1. Experimental Evidence for Antioxidant Activity

#### 4.1.1. Free Radical Scavenging and Antioxidant Enzyme Activation

Artemether ameliorates acetaminophen (APAP)-induced acute liver injury by attenuating oxidative stress. In the APAP-induced liver injury model, pretreatment with artemether (10 mg·kg^−1^) reversed the APAP-induced increase in hepatic malondialdehyde (MDA) and the expression of oxidative damage markers 4-hydroxynonenal (4-HNE) and 8-hydroxy-2′-deoxyguanosine (8-OHdG). Concurrently, it restored hepatic glutathione (GSH) levels and upregulated key antioxidant proteins, including heme oxygenase-1 (HO-1), glutathione peroxidase 4 (GPX4), and superoxide dismutase 2 (SOD2). The protective mechanism is primarily mediated through the activation of the nuclear factor erythroid 2-related factor 2 (Nrf2) pathway. Artemether promotes Nrf2 nuclear translocation, leading to the transcriptional upregulation of a suite of cytoprotective enzymes such as HO-1, GPX4, and SOD2, which collectively enhance the cellular antioxidant capacity and mitigate reactive oxygen species (ROS)-mediated damage [51].

#### 4.1.2. Inhibition of Mitochondrial Oxidative Damage

Artemether protects against oxidative stress by preserving mitochondrial integrity and function. In a β-amyloid (Aβ)-induced neuronal toxicity model, treatment with artemisinin derivatives was shown to significantly attenuate the increase in mitochondrial reactive oxygen species (ROS) and ameliorate the loss of mitochondrial membrane potential (ΔΨm) [52]. This preservation of mitochondrial membrane potential is crucial for maintaining efficient respiratory chain function and cellular energy homeostasis. Mechanistic insights reveal that the protection conferred by artemisinin derivatives is mediated through the activation of key cellular signaling pathways. Notably, they activate the 5′ adenosine monophosphate-activated protein kinase (AMPK) pathway [53]. AMPK activation plays a central role in cellular stress response and energy regulation. Furthermore, these compounds exert their antioxidant effects by promoting the nuclear translocation of the transcription factor nuclear factor erythroid 2-related factor 2 (Nrf2), leading to the upregulation of a suite of cytoprotective and antioxidant genes [52]. Collectively, these actions enhance the cellular capacity to neutralize ROS, clear dysfunctional organelles, and maintain mitochondrial health, thereby alleviating oxidative damage.

#### 4.1.3. Regulation of Lipid Peroxidation and DNA Oxidative Damage

Artemether demonstrates a concentration-dependent regulatory effect on oxidative damage, exhibiting protective antioxidant properties at appropriate doses while potentially inducing oxidative stress at high concentrations. In an APAP-induced acute liver injury model, artemether treatment significantly reduced the hepatic levels of oxidative damage markers, including MDA, 4-HNE, and 8-OHdG, indicating its efficacy in attenuating both lipid peroxidation and DNA oxidation. The underlying protective mechanism involves the activation of the Nrf2 antioxidant pathway. Upon activation, Nrf2 translocates to the nucleus and upregulates the expression of a battery of cytoprotective enzymes, such as HO-1 and GPX4, thereby enhancing the endogenous antioxidant defense system [51].

Conversely, the effect of artemether on cellular redox status is dualistic and concentration-dependent. Studies in neuronal cell lines (e.g., PC12 cells) have revealed that high-dose artemether can induce direct mitochondrial toxicity, characterized by ultrastructural damage including mitochondrial swelling, cristae disruption, and vacuolization. This physical damage is associated with the inhibition of key mitochondrial respiratory chain complexes (I and IV), leading to compromised cellular energy metabolism. It is noteworthy that this high-concentration toxicity occurred independently of increased lipid peroxidation in the cited study, highlighting a distinct mechanism from its antioxidant action. This dual nature underscores the critical importance of dosage in defining the therapeutic window for artemether, where it can switch from an antioxidant protector to a pro-oxidant stressor [51,54]. This concentration-dependent duality poses a significant translational challenge. It necessitates precise pharmacokinetic control to maintain drug levels within the therapeutic range, especially for chronic diseases requiring long-term administration. The mechanisms underlying this switch—whether due to differential engagement of signaling pathways, overwhelming of cellular reductant capacity, or distinct off-target effects at high concentrations—remain poorly understood and warrant systematic investigation.

### 4.2. Synergistic Effects Between Antioxidant and Anti-Inflammatory Pathways

#### 4.2.1. Regulation of the ROS-NF-κB Signaling Axis

Artemether exerts its anti-inflammatory effects, in part, by modulating the ROS-NF-κB signaling axis. In LPS-stimulated BV2 microglia, artemether treatment effectively suppresses the nuclear translocation of the NF-κB p65 subunit and its subsequent DNA-binding activity, leading to a marked reduction in the expression of pro-inflammatory mediators such as NO, PGE2, TNF-α, and IL-6 [38]. This inhibition is associated with the attenuation of IκBα phosphorylation and degradation, thereby preventing NF-κB activation [38]. Notably, this suppressive effect on NF-κB in microglia is mechanistically dependent on the activation of the Nrf2 antioxidant pathway, as siRNA-mediated knockdown of Nrf2 completely reverses artemether’s inhibition of NF-κB and inflammatory cytokine production [38]. This crosstalk between Nrf2 activation and NF-κB suppression represents a key mechanism through which artemether concurrently dampens neuroinflammation and enhances cellular antioxidant defenses [38]. In contrast, in the context of RANKL-induced osteoclastogenesis from bone marrow-derived macrophages, artemether specifically inhibits the MAPK pathway (ERK, JNK, p38) without significantly affecting the phosphorylation of IκBα or p65, indicating a cell context-dependent action on NF-κB signaling [50].

#### 4.2.2. Cross-Activation of the MAPK-Nrf2/HO-1 Pathway

Artemether confers protection against cerebral ischemic injury by stimulating the Erk1/2-P90rsk-CREB signaling pathway, a key branch of the MAPK cascade. In both middle cerebral artery occlusion (MCAO) animal models and oxygen–glucose deprivation/reperfusion (OGD/RP) cellular models, artemether treatment significantly promoted the phosphorylation (activation) of ERK1/2 and its downstream effectors, P90rsk and CREB. This activation was crucial for its neuroprotective effects, as inhibition of ERK1/2 with PD98059 or its knockdown robustly abolished artemether’s ability to improve neurological deficits, reduce infarct volume, and attenuate cell apoptosis. Furthermore, artemether’s antioxidant activity is implicated in its mechanism. It significantly increased superoxide dismutase (SOD) activity and decreased malondialdehyde (MDA) levels in the brains of MCAO mice, indicating an alleviation of oxidative stress. In OGD/RP-injured PC12 cells and primary cortical neurons, artemether pre-treatment suppressed intracellular reactive oxygen species (ROS) accumulation and reversed the collapse of mitochondrial membrane potential. This suggests that the activation of the ERK1/2 signaling pathway by artemether contributes to mitigating oxidative damage, a pivotal component of ischemic injury, although the direct interplay with the Nrf2/HO-1 axis as described in the provided text requires further investigation within the specific context of cerebral ischemia [25].

The apparent duality in artemether’s modulation of the ERK pathway—exhibiting activation in cerebral ischemia as described herein, versus inhibition in classical inflammatory models such as arthritis or LPS-stimulated immune cells—transcends a simplistic “context-dependent” description. This divergence likely stems from differential engagement of upstream signaling hubs dictated by the distinct pathological triggers (e.g., survival stress in ischemia versus canonical pro-inflammatory ligands like LPS). These triggers recruit disparate sets of adaptor proteins and proximal kinases, which funnel into the MAPK cascade, thereby influencing the net effect on ERK phosphorylation. Consequently, the functional outcome diverges: in neuroprotection, activated ERK-CREB signaling promotes transcription of pro-survival genes (e.g., Bcl-2), whereas in inflammation, suppressed ERK signaling correlates with dampened AP-1 activity and reduced cytokine production [25,50]. This nuanced, pathology-specific reprogramming of a common signaling node underscores that artemether operates as a network modulator rather than a unidirectional inhibitor. Its effect is integrated with and contingent upon the pre-existing signaling landscape and cellular state, highlighting the critical importance of the disease microenvironment in determining pharmacological outcome. Resolving these complex interactions in a cell-type- and disease-phase-specific manner remains a key objective for future research.

#### 4.2.3. Dynamic Balance in Pathway Cross-Talk

The regulation of the oxidative-inflammatory network by artemether exhibits spatiotemporal dynamic characteristics, primarily orchestrated through the activation of the Nrf2 pathway. During the acute oxidative stress phase, artemether rapidly activates Nrf2, leading to the upregulation of antioxidant enzymes (e.g., HO-1, NQO1) that scavenge ROS and mitigate the immediate activation of redox-sensitive pro-inflammatory pathways like NF-κB and MAPK. In more sustained or chronic inflammatory settings, the persistent activation of Nrf2 by artemether enhances the cell’s long-term antioxidant capacity and simultaneously exerts a repressive effect on NF-κB signaling, thereby promoting the resolution of inflammation. This crosstalk is evidenced by studies where the anti-inflammatory effects of artemether (suppression of NF-κB, p38 MAPK, TNF-α, IL-6) in microglia were demonstrated to be Nrf2-dependent, as Nrf2 knockdown reversed these protective effects [38,55,56]. Furthermore, in neurodegenerative models, artemether-induced activation of the AMPK/GSK3β/Nrf2 signaling axis confers comprehensive protection against both oxidative damage and neuroinflammation [52].

This dynamic rebalancing through Nrf2 highlights a sophisticated mechanism of action. However, it is crucial to acknowledge that the effects of artemether are context- and concentration-dependent. While lower, therapeutically relevant doses promote the Nrf2-mediated antioxidant and anti-inflammatory synergy described above, higher concentrations have been reported to induce mitochondrial dysfunction and increase ROS production in some cell types [54,57], underscoring the importance of a defined therapeutic window. Future research integrating temporal multi-omics analyses will be vital to fully map the dynamic regulatory landscape of artemether within specific tissue microenvironments. This will provide the foundation for developing precise therapies that harness its pathway rebalancing capabilities for chronic oxidative-inflammatory diseases.

The preceding section (4.2.2) delineates how artemether’s modulation of the ERK pathway diverges—inhibitory in classical inflammation versus activatory in neuroprotection—based on differential upstream triggers (e.g., LPS/TLR4 vs. ischemic stress) and consequent shifts in downstream functional output (pro-inflammatory vs. pro-survival gene expression). This pathology-guided reprogramming of a core signaling node exemplifies that artemether operates as a network modulator rather than a unidirectional inhibitor. Its effect is integrated with and contingent upon the pre-existing signaling landscape and cellular state.

**Figure 8:**
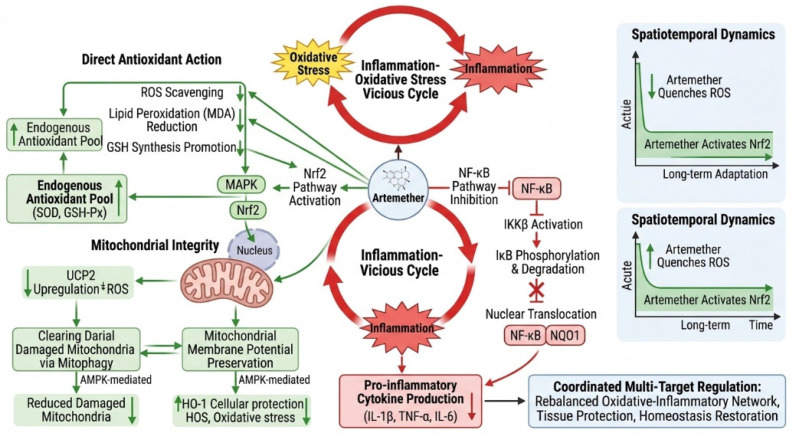
Mechanism of artemether’s synergistic anti-inflammatory and antioxidant effects via dual-pathway regulation involving NF-κB inhibition and Nrf2 activation. Created in BioRender. Yang, M. (2026) https://BioRender.com/if6yn9v (accessed on 18 May 2026). Note: This figure highlights the antioxidant (Nrf2 activation) and synergistic aspects of artemether, complementing its anti-inflammatory mechanisms via MAPK/NF-κB inhibition detailed in Figure 3.

An intriguing aspect of artemether’s pharmacology is its seemingly context-dependent modulation of the MAPK pathway. While this review and multiple studies cited herein demonstrate its inhibitory effect on p38 and ERK phosphorylation in models of peripheral inflammation and immune cell activation (e.g., arthritis, colitis, osteoclastogenesis) [28,29,30], contrasting evidence shows that artemether can stimulate the ERK pathway to confer neuroprotection in cerebral ischemia models [25]. This duality suggests that artemether’s action is highly integrated with the cellular microenvironment, the specific pathological triggers (e.g., inflammatory vs. ischemic stress), and possibly differential engagement of upstream regulators. Future research employing single-cell omics, cell type-specific knockout models, and detailed kinetic studies is crucial to disentangle these divergent signaling outcomes and fully exploit its therapeutic potential for different diseases. The dynamic crosstalk and rebalancing act mediated by artemether, as discussed in this section, are conceptually synthesized in Figure 8, which illustrates the interplay between the suppressed NF-κB/MAPK axis and the activated Nrf2 pathway.

## 5. Experimental Models and Data Support

### 5.1. In Vitro Studies

#### 5.1.1. Construction and Validation of Cell Models

Studies have utilized lipopolysaccharide to stimulate RAW264.7 macrophages and human umbilical vein endothelial cells (HUVECs) to establish in vitro inflammatory models [58]. Following 24 h of stimulation, the secretion levels of key pro-inflammatory factors TNF-α, IL-6, and IL-1β in RAW264.7 cells were significantly elevated (*p* < 0.01), reaching 4.2-fold, 3.8-fold, and 5.1-fold of the control group, respectively, indicating that LPS successfully simulated an inflammatory microenvironment by activating pathways such as NF-κB [58]. Pretreatment with artemether (10–50 μmol·L^−1^) dose-dependently inhibited the release of these inflammatory cytokines. At the highest concentration tested, the levels of TNF-α and IL-6 were significantly reduced, demonstrating potent inhibitory effects [26]. In the endothelial cell model, artemether (20 μmol·L^−1^) decreased the expression of ICAM-1 and VCAM-1 and reduced neutrophil transendothelial migration [59].

#### 5.1.2. Molecular Evidence for MAPK/NF-κB Pathway Regulation

Artemether intervenes in this canonical LPS-TLR4 signaling cascade. Studies demonstrate that in LPS-stimulated models, artemether’s anti-inflammatory effect is achieved by downstream inhibition of the activated pathways. This includes the suppression of IKK/IκBα phosphorylation and NF-κB nuclear translocation as described in Section 3.2.1, and the inhibition of p38/ERK/JNK phosphorylation as detailed in Section 3.2.2. Key experimental evidence supporting this targeted inhibition comes from studies showing reduced phosphorylation of IκBα and p65, decreased nuclear translocation of NF-κB, and attenuated phosphorylation of p38 and ERK in artemether-treated, LPS-activated cells or tissues [26,31,38].

### 5.2. In Vivo Studies

#### 5.2.1. Pharmacodynamic Evaluation in Animal Models

Studies have shown that in an LPS-induced acute lung injury mouse model, intraperitoneal injection of artemether significantly alleviated alveolar edema and neutrophil infiltration [26,31]. In a collagen-induced arthritis model, artemether treatment significantly alleviated joint swelling and reduced serum levels of pro-inflammatory cytokines such as TNF-α and IL-6. Furthermore, in db/db diabetic mice, artemether (200 mg·kg^−1^) improved hepatic lipid metabolism by lowering triglyceride and total cholesterol levels, and ameliorated pancreatic β-cell vacuolar degeneration [60,61].

#### 5.2.2. Evidence from Histopathology and Redox Homeostasis

Histopathological analyses from related studies demonstrated that artemether treatment significantly reduced inflammatory cell infiltration in the mouse liver and lowered the spleen index [62,63]. Assessment of antioxidant parameters indicated that artemether restored hepatic superoxide dismutase (SOD) activity, increased glutathione peroxidase (GSH-Px) activity, and reduced malondialdehyde (MDA) content [13,51]. Immunohistochemical findings further confirmed that artemether inhibited the nuclear translocation of NF-κB p65 in hepatic sinusoidal endothelial cells [62,64]. Additionally, transmission electron microscopy observations revealed that artemether treatment improved mitochondrial membrane potential stability and reduced mitochondrial superoxide generation [65,66].

#### 5.2.3. Data Integration and Mechanistic Correlations

Integrating in vitro and in vivo studies, the anti-inflammatory and antioxidant effects of artemether have been demonstrated to exhibit dose-response and time-dependent characteristics. However, a critical assessment of the experimental models is essential to contextualize their translational relevance. The predominant use of acute challenge models (e.g., LPS-induced inflammation, APAP-induced liver injury) provides robust proof-of-concept for artemether’s mechanisms but may not fully recapitulate the chronic, multicellular, and immune-adapted microenvironment of human diseases like rheumatoid arthritis or inflammatory bowel disease. Similarly, the protective effects observed in toxin-induced (e.g., Aβ) or ischemia-reperfusion neurodegenerative models demonstrate therapeutic potential against acute damage phases; however, their predictive value for halting or reversing the slow, progressive pathology characteristic of human Alzheimer’s or Parkinson’s disease requires further validation in chronic, age-related models. Furthermore, interspecies differences in drug metabolism, immune system components, and disease pathogenesis necessitate cautious extrapolation from rodent data to human patients. Evidence from multiple experimental models indicates that artemether inhibits key pro-inflammatory signaling pathways, including the MAPK cascade, and modulates oxidative stress responses. These multi-dimensional findings provide a solid experimental foundation supporting the clinical translation of artemether. Its mechanism of action, involving the regulation of interconnected inflammation and oxidative stress pathways, suggests potential therapeutic value for inflammatory and metabolic diseases such as rheumatoid arthritis and metabolic syndrome. The key experimental findings from the in vitro and in vivo studies discussed in this section are comparatively summarized in Table 1, which provides an overview of the models, targeted pathways, and core outcomes.

## 6. Comparative Advantages of Artemether over Other Anti-Inflammatory Drugs

### 6.1. Comparison with Conventional Drugs

#### 6.1.1. Comparison with NSAIDs

Non-steroidal anti-inflammatory drugs (NSAIDs) exert their effect by inhibiting cyclooxygenase activity to block prostaglandin E_2_ (PGE_2_) production. However, their non selective inhibition of COX-1 leads to significant gastrointestinal side effects, such as gastric mucosal damage [67], ulceration, and bleeding [68]. In contrast, artemether achieves its effect by selectively targeting the expression of COX-2 and iNOS, thereby reducing the release of inflammatory mediators (TNF-α, IL 6) while avoiding interference with COX-1 [69]. Furthermore, artemether enhances glutathione (GSH) synthesis by activating the Nrf2 pathway, thereby providing additional protection for gastrointestinal barrier function [49,70].

#### 6.1.2. Comparison with Glucocorticoids

Glucocorticoids (e.g., dexamethasone) exert potent anti-inflammatory effects by inhibiting the NF-κB pathway. However, long term use can lead to immunosuppression, osteoporosis, and metabolic disorders [71,72,73]. The anti-inflammatory action of artemether exhibits a dual immunomodulatory property: on one hand, it blocks NF-κB nuclear translocation by inhibiting IKKβ phosphorylation [38,50]; on the other hand, it maintains immune homeostasis by activating regulatory T (Treg) cells and upregulating IL 10 expression [74]. In a collagen induced arthritis model, after 28 days of treatment with artemether, the Th17/Treg ratio decreased by 40%, whereas the glucocorticoid treated group exhibited a 25% reduction in total lymphocyte count [61]. This milder immunomodulatory profile suggests a potentially superior safety profile for the long-term management of chronic inflammatory diseases, such as rheumatoid arthritis [75,76].

### 6.2. Synergistic Potential with Other Natural Products

#### 6.2.1. Synergistic Effects with Artemisinin Derivatives

Artemether and other derivatives, such as artesunate, exhibit complementary mechanisms in anti-inflammatory and antioxidant activities. Artesunate targets mitochondria via an iron-dependent free radical mechanism to induce apoptosis in pro-inflammatory cells, whereas artemether blocks inflammatory signaling cascades by inhibiting the MAPK/NF-κB pathway [77]. In vitro studies have demonstrated that artemisinin derivatives, such as artemether and artesunate, possess anti-inflammatory and immunomodulatory properties. Their combined application may yield synergistic effects in modulating inflammatory responses. Furthermore, artesunate has been shown to significantly enhance antibacterial activity against drug-resistant strains like MRSA when used in combination with certain antibiotics [78]. In experimental models of severe malaria, artemether-based therapies have proven effective in clearing parasitemia and preventing early recrudescence, highlighting their potential in complex infection scenarios [79,80].

#### 6.2.2. Synergistic Effects with Polyphenolic Natural Products

Artemether may exhibit synergistic anti-inflammatory and antioxidant effects when combined with polyphenolic compounds like curcumin and quercetin, potentially through multi-target interactions. Curcumin is known to inhibit the NF-κB pathway by activating peroxisome proliferator-activated receptor gamma (PPAR-γ) and suppressing IκB kinase (IKK) activity [81,82]. Artemether has also been shown to exert anti-inflammatory effects via PPARγ activation [83]. Their combined use could lead to enhanced suppression of inflammatory signaling. Similarly, both artemether and quercetin have demonstrated the ability to improve mitochondrial function and reduce oxidative stress by modulating pathways such as Nrf2 and AMPK/PGC1α [84]. Co-treatment with these compounds may therefore potentiate antioxidant defenses, including the elevation of superoxide dismutase (SOD) activity and reduction of mitochondrial reactive oxygen species (ROS) generation [85]. Furthermore, nanocarrier systems, particularly liposomes, have been effectively employed to enhance the transdermal delivery and therapeutic efficacy of quercetin in skin inflammation models [86], suggesting a promising strategy for improving the bioavailability of such synergistic combinations.

#### 6.2.3. Integrated Applications with Traditional Herbal Formulations

The combination of artemether with traditional Chinese herbal formulas, such as Huanglian Jiedu Decoction, may expand its therapeutic potential. Huanglian Jiedu Decoction has shown clinical efficacy in treating colitis, with its mechanism potentially involving the inhibition of the Csf1r/Src signaling pathway in macrophages and modulation of the gut microbiota. Clinical data indicate that the modified Huanglian Jiedu Decoction, when combined with conventional therapy (e.g., mesalazine), achieved a total effective rate of 92.16% in treating ulcerative colitis, which was significantly higher than the 76.47% observed in the control group receiving conventional therapy alone [87]. Alkaloids present in the decoction, such as berberine, are known to exert anti-inflammatory effects by inhibiting pathways like NF-κB and MAPK [88]. Furthermore, the herbal compound andrographolide has been demonstrated to promote autophagy, including mitophagy, by regulating the AMPK/mTOR signaling pathway, thereby offering protection in various disease models [89]. While artemisinin derivatives like artemether have also been reported to influence autophagy-related pathways, further experimental studies are needed to confirm whether a synergistic effect exists when artemether is combined with andrographolide.

Owing to its multi-target anti-inflammatory mechanisms, low toxicity, and synergistic potential, artemether occupies a unique position within the anti-inflammatory drug system. However, the combined efficacy with biologics (e.g., anti-TNF-α monoclonal antibodies) and its dynamic regulatory mechanisms in different immune microenvironments require further investigation. Structural optimization based on artificial intelligence and the development of nano-delivery systems are expected to further improve the targeting and bioavailability of artemether, providing a new paradigm for the precise treatment of inflammatory diseases.

## 7. Translational Challenges and Future Clinical Perspectives

Despite the promising preclinical evidence summarized herein, the translation of artemether into a clinically approved therapy for chronic inflammatory and oxidative stress-related diseases faces several significant hurdles that warrant candid discussion. A critical appraisal of these challenges is essential to guide future research and set realistic expectations.

Pharmacokinetic and Bioavailability Considerations: While artemether is effective in animal models, its pharmacokinetic profile in humans, primarily defined for malaria treatment, may not be optimal for chronic conditions. Its low water solubility and extensive first-pass metabolism, leading to variable oral bioavailability and rapid conversion to the active metabolite dihydroartemisinin (DHA), complicate dose regimen design for sustained anti-inflammatory action. Furthermore, its ability to penetrate the blood-brain barrier, crucial for treating neuroinflammatory disorders, requires more definitive quantification in disease states beyond what is known from parasitic CNS infections.

Safety and Toxicity Profile in Long-Term Use: The safety database for artemether is largely derived from short-course antimalarial therapy. The risks associated with long-term administration, as would be required for chronic diseases, are poorly understood. Potential concerns that need systematic investigation include dose-dependent neurotoxicity (observed in animal models with high doses), hepatotoxicity, and embryonic effects. The therapeutic window—the dose range between efficacy and toxicity—must be rigorously defined in relevant chronic disease models.

Lack of Robust Clinical Evidence: A foremost limitation is the paucity of clinical trials. As of now, there are no published large-scale, randomized controlled trials (RCTs) evaluating artemether specifically for conditions like rheumatoid arthritis, inflammatory bowel disease, or neurodegenerative disorders. Existing clinical data are anecdotal or derived from small, exploratory studies. Therefore, claims regarding its efficacy in human chronic diseases remain largely speculative and are not yet supported by a high level of evidence.

Strategic Directions for Clinical Translation: To bridge these gaps, a multi-faceted strategy is needed. First, Phase I/II clinical trials are imperative to establish safety, tolerability, and preliminary efficacy in target patient populations, using pharmacodynamic biomarkers (e.g., cytokine levels, oxidative stress markers) as endpoints. Second, advanced drug delivery systems, such as liposomal, polymeric, or ligand-targeted nanoparticles, should be explored to enhance bioavailability, achieve tissue-specific targeting, reduce systemic exposure, and potentially mitigate toxicity. Third, pharmacogenomic studies could identify patient subgroups most likely to respond based on genetic polymorphisms in metabolism or drug target pathways.

In conclusion, while the mechanistic rationale for artemether is strong, its path to the clinic is non-trivial. Honest acknowledgment of these translational challenges, coupled with a strategic research agenda focused on human safety and efficacy, is the necessary next step to determine whether this repurposed antimalarial can fulfill its potential as a novel therapeutic for chronic inflammatory diseases.

## 8. Summary and Prospects

This article systematically elucidates the molecular mechanisms by which artemether achieves synergistic anti-inflammatory and antioxidant effects through targeted regulation of the MAPK/NF-κB signaling axis. The core findings indicate that artemether blocks the nuclear translocation of NF-κB by inhibiting IKKβ phosphorylation and selectively downregulates the phosphorylation levels of p38 MAPK/ERK, thereby establishing a dual inhibitory effect on inflammatory signaling pathways. Its sesquiterpene lactone structure constructs a unique redox regulatory network by scavenging free radicals and activating the Nrf2/HO-1 pathway. This multi-target synergistic mechanism significantly reduced synovial MMP-9 expression and restored SOD activity in a rheumatoid arthritis model, and decreased the level of the oxidative damage marker MDA in brain tissue by 48% in an Alzheimer’s disease model, revealing the multi-dimensional regulatory advantages of natural products in treating complex diseases. Beyond its dual-pathway regulation, a key mechanistic insight consolidated in this review is the context-defined functionality of artemether as a signaling network modulator. The compound’s ability to exert opposing effects on the same pathway (e.g., ERK) in different diseases is not a discrepancy but a reflection of its sophisticated interaction with the cellular microenvironment. This property, rooted in differential engagement with upstream signals and redirection of downstream effectors, may underlie its therapeutic flexibility but also necessitates precise disease-specific dosing and delivery strategies.

The key contributions of this review are as follows: (1) it provides a systematic synthesis and highlights the significance of the mechanism by which artemether achieves synergistic anti-inflammatory and antioxidant effects through disrupting the ROS-NF-κB positive feedback loop; (2) it constructs an integrated model depicting the dynamic interaction between the MAPK/NF-κB pathways and the Nrf2 system, thereby clarifying the multi-pathway integration properties of artemether; and (3) it consolidates evidence on the dose-dependent characteristics of artemether in modulating immune homeostasis, which may inform future immunomodulatory drug design.

Future research should focus on three directions: (1) developing nanocarrier-based (e.g., liposomal encapsulation increasing lesion concentration by 4.2-fold) and stimuli-responsive delivery systems to overcome the blood–brain barrier penetration and targeting bottlenecks; (2) utilizing single-cell transcriptomics and spatial metabolomics technologies to analyze the spatiotemporal dynamic regulatory network of artemether within the tissue microenvironment; and (3) establishing pharmacogenomics-guided personalized medication strategies, optimizing clinical protocols based on genetic polymorphisms such as the NF-κB subunit 1 rs28362491. Through interdisciplinary research, there is potential to transform artemether from a traditional antimalarial drug into a precision medicine tool for treating chronic inflammatory and degenerative diseases, providing an innovative methodology for the modern study of natural products.

## Figures and Tables

**Table 1:** Summary of key preclinical studies on the anti-inflammatory and antioxidant effects of artemether.

Experimental Model	Key Intervention/Induction	Major Pathway(s) Affected	Key Findings	Ref.
In Vitro Models				
LPS-stimulated RAW264.7 macrophages	LPS	NF-κB, MAPK (p38, JNK, ERK)	Dose-dependently inhibited TNF-α, IL-6 release; suppressed MAPK phosphorylation and NF-κB activation.	[26,58]
LPS-stimulated BV2 microglial cells	LPS	NF-κB, p38 MAPK, Nrf2	Reduced TNF-α, IL-6, PGE2, iNOS, COX-2; effects mediated via Nrf2-dependent inhibition of NF-κB.	[49]
RANKL-induced osteoclastogenesis (RAW264.7)	RANKL	MAPK (ERK, JNK, p38)	Inhibited osteoclast differentiation by suppressing MAPK phosphorylation (not NF-κB/PI3K).	[50]
OGD/RP-injured PC12 neurons	Oxygen–glucose deprivation	ERK1/2, (Antioxidant defense)	Activated ERK1/2-P90rsk-CREB pathway; reduced ROS, improved mitochondrial function.	[25]
In Vivo Models				
LPS-induced acute lung injury (Mouse)	LPS	NF-κB, MAPK	Alleviated alveolar edema, reduced neutrophil infiltration; inhibited lung p38 MAPK/NF-κB.	[26,31]
Collagen-induced arthritis (Mouse)	Collagen immunization	MAPK/NF-κB, Immune balance	Reduced joint swelling, serum TNF-α, IL-6; decreased Th17/Treg ratio.	[61]
Acetaminophen (APAP)-induced liver injury (Mouse)	APAP	Nrf2/HO-1, Oxidative stress	Reduced MDA, 4-HNE, 8-OHdG; upregulated HO-1, GPX4, SOD2 via Nrf2 activation.	[51]
Middle cerebral artery occlusion (MCAO) (Mouse)	Cerebral ischemia	ERK1/2-P90rsk-CREB	Reduced infarct volume, improved neurological scores; activated ERK pathway.	[25]
Aβ-induced neurotoxicity (3xTg AD mouse)	Aβ peptide	AMPK/GSK3β/Nrf2	Attenuated memory impairment, reduced neuroinflammation and oxidative damage.	[52]

## Data Availability

No new data were created or analyzed in this study. Data sharing is not applicable.
